# Regulatory mechanism for host-cell contact-dependent T3SS gene expression in *Vibrio parahaemolyticus*

**DOI:** 10.1128/msystems.00251-25

**Published:** 2025-06-17

**Authors:** Sarunporn Tandhavanant, Hiroyuki Terashima, Hirotaka Hiyoshi, Dhira Saraswati Anggramukti, Nopadol Precha, Tetsuya Iida, Shigeaki Matsuda, Narisara Chantratita, Toshio Kodama

**Affiliations:** 1 Department of Bacteriology, Institute of Tropical Medicine, Nagasaki University, Nagasaki, Japan; 2 Department of Microbiology and Immunology, Faculty of Tropical Medicine, Mahidol University 115374, Bangkok, Thailand; 3 Department of Pharmacology, College of Pharmacy, Kinjo Gakuin University 12873, Nagoya, Japan; 4 Department of Bacterial Infections, Research Institute for Microbial Diseases, Osaka University 12936 , Osaka, Japan; 5 Department of Environmental Health and Technology, School of Public Health, Walailak University 722944, Nakhon Si Thammarat, Thailand; National Institute of Allergy and Infectious Diseases, Bethesda, Maryland, USA

**Keywords:** *Vibrio parahaemolyticus*, type III secretion system, gene regulation, gatekeeper

## Abstract

Many pathogenic bacteria regulate gene expression in response to the surrounding environment to establish infection. One such mechanism is the regulation of gene expression in response to contact with host cells. Here, we show that *Vibrio parahaemolyticus*, a causative agent of foodborne gastroenteritis, has a host-cell contact-dependent regulatory mechanism for virulence gene expression. Type III secretion system 2 (T3SS2), an essential virulence determinant for acute gastroenteritis encoded by *V. parahaemolyticus* pathogenicity island (Vp-PAI), recognizes host-cell contact by sensing high intracellular K^+^ levels and switching its secretory substrates. The switching of secretory substrates is regulated by proteins called gatekeepers. Mutants deficient in the gatekeeper genes lose the ability to switch secretory substrates and lock their secretory state into a host-cell contact-dependent manner. Transcriptomic analysis of these mutants revealed the upregulation of Vp-PAI genes, which was dependent on T3SS2 secretory activity, suggesting the presence of a negative regulator secreted by T3SS2. Comparative proteomic analyses identified a previously unrecognized T3SS2 secretory substrate, VPA1369 (VtrN), that negatively regulates Vp-PAI gene transcription. Secretion of VtrN was promoted under conditions that mimic host-cell contact. *vtrN* gene deletion specifically upregulated Vp-PAI gene expression, independent of T3SS2 secretory activity, indicating its role as a repressor. VtrN interacts with VtrB, a key transcription factor for Vp-PAI genes, suppressing its transcriptional activity. This mechanism illustrates how *V. parahaemolyticus* enhances virulence gene expression upon host-cell contact through the T3SS2 recognition system, highlighting an adaptive strategy for establishing infection.

**IMPORTANCE:**

The type III secretion system (T3SS) is a crucial virulence factor tightly regulated for optimal host manipulation and virulence. This study revealed that the expression of T3SS2, a key virulence factor in *Vibrio parahaemolyticus* that causes acute gastroenteritis, is strictly regulated by host-cell contact. VtrN, a negative regulator exported from the bacterium through T3SS2, plays a key role in this host-cell contact-dependent gene transcriptional process. VtrN binds directly to the master regulator of Vp-PAI, the region encoding T3SS2, and represses its transcriptional activity. Upon host-cell contact, VtrN export is promoted, leading to the derepression of Vp-PAI gene expression. Thus, *V. parahaemolyticus* can effectively upregulate the expression of virulence factors when interacting with the host cells. Understanding these regulatory mechanisms could lead to innovative infection control strategies, opening new avenues for research and discovery.

## INTRODUCTION

Type III secretion system (T3SS) is an injectosome and key virulence factor found in many gram-negative symbionts and pathogens with diverse evolution (1). Environmental sensing in response to temperature, salt concentrations, biological compounds, and chemical substances triggers global transcriptional regulators that influence T3SS expression (2). To maximize their survival within the host, bacteria have evolved a sophisticated mechanism to tightly control the T3SS via master transcriptional regulators and by recognizing signals associated with host-cell contact (3, 4). Intracellular Ca^2+^, H^+^, and K^+^ concentrations are recognized by T3SSs of *Pseudomonas aeruginosa* and *Yersinia* spp., SPI-2 of *Salmonella* spp., and T3SS2 of *Vibrio parahaemolyticus,* as host-cell contact signals (5–8). Sensing host-cell contact by gatekeepers of injectosome stimulates the last step of T3SS secretion, leading to a shift in secretory substrates from translocators to effectors (9). Additionally, host-cell contact activates T3SS-related gene transcription, which is affected by the secretion of T3SS effectors and negative regulators.

Some mechanisms underlying the host-cell contact-dependent regulation of gene expression have been reported for several T3SSs. In *P. aeruginosa*, host-cell contact alters interactions between T3SS regulatory proteins ExsACDE (10, 11). In a steady state, master transcriptional regulator ExsA is coupled with inhibitor ExsD, and effector ExsE and its chaperone ExsC are bound. In the host-cell contact state, by sensing the low Ca^2+^ of the host cells through T3SS, ExsC releases ExsE and promotes export through T3SS. ExsC also acts as an anti-inhibitor of this regulatory system. Free ExsC sequesters ExsD away from ExsA, triggering T3SS cluster transcription (10, 11). In *Yersinia* spp., the LcrQ-LcrH-YopD complex suppresses the expression of master regulator *lcrF* by recruiting RNase complex to terminate mRNA (12). YopD export from *Yersinia* disrupts the complex after T3SS senses low Ca^2+^. As a result, stable *lcrF* mRNA levels increase T3SS expression (12).

In some T3SSs, host-cell contact promotes the export of transcriptional inhibitors. In *Shigella flexneri*, anti-activator OspD1 forms a complex with Spa15 and MxiE, thereby blocking transcriptional regulator MxiE under non-inducing conditions (13). Host-cell contact activates the *S. flexneri* T3SS, triggering the export of OspD1 and releasing the chaperone protein IpgC, which in turn activates the transcription of effector genes via MxiE (13, 14). The negative regulator BtrA/BspR acts as an antagonist of BtrS, a BvgAS-regulated extracytoplasmic function sigma factor that regulates T3SS expression in *Bordetella* (15). BtrA/BspR is exported from bacteria through the T3SS in response to iron depletion, increasing T3SS expression (16). The signaling involved in contact-dependent gene expression ultimately converges on the T3SS master regulator activation (3, 4). However, the pathways leading to this activation are diverse, and the detailed mechanisms remain unclear, including *V. parahaemolyticus* T3SS2.

*V. parahaemolyticus* spreads worldwide via inshore marine water and contaminates seafood products (17), causing acute gastroenteritis. Clinical *V. parahaemolyticus* contains two chromosomes, each harbors distinct T3SS gene clusters, referred to as T3SS1 and T3SS2 (18). An 80kb pathogenic island encoding T3SS2, known as the *V. parahaemolyticus* pathogenicity island (Vp-PAI), is essential for enterotoxicity in various animal models (19, 20). VtrA, VtrC, and VtrB regulate T3SS2 expression under specific conditions (21, 22). Adequate salt concentration and temperature in seawater activate H-NS release from the promoter of *vtrB*, T3SS2 master regulator (23). ToxR, which acts in a cell density-dependent manner (24), positively regulates transcription of *vtrA* and *vtrB* (25, 26). Bile acids, present in the host intestinal environment, initiate T3SS2 expression through the VtrA-VtrB transcriptional regulatory system (21, 27). In addition to changes in gene expression driven by environmental stimuli, T3SS2 modulates secretory activity in a host-cell contact-dependent manner (8). In a previous study, we identified gatekeepers, VgpA and VgpB, as factors involved in this modulation. VgpA and VgpB suppress effector secretion before contact with the host cells. Host-cell contact indicated by sensing high intracellular K^+^ facilitates VgpA and VgpB secretions. This process relieves the repression of effector secretion by VgpA and VgpB, thereby promoting effector injection (8).

T3SS secretion occurs in successive steps, with gatekeepers regulating the switch between translocators and effectors, which is thought to mimic the state of bacterial contact with the host cell. Deleting the gatekeeper gene leads to irregular secretion, resulting in spontaneous secretion of effectors instead of translocators (28–31). Host-cell contact-dependent gene regulatory mechanisms have been reported for several T3SSs (10–16). However, whether *V. parahaemolyticus* T3SS2 exhibits this mechanism is not entirely known. This study investigated whether this organism has a host-cell contact-dependent gene expression regulatory mechanism. We hypothesized that analysis of gene expression changes caused by deleting gatekeeper genes, *vgpA* and *vgpB*, would unveil host-cell contact-dependent T3SS2 expression changes in *V. parahaemolyticus*. Our results revealed that an unrecognized T3SS2-dependent secreted protein regulates the activity of a master transcriptional regulator that activates T3SS2 gene transcription in correlation with host-cell contact.

## RESULTS

### Deleting the gatekeeper genes for T3SS2 led to upregulation of the Vp-PAI gene

**Fig 1:**
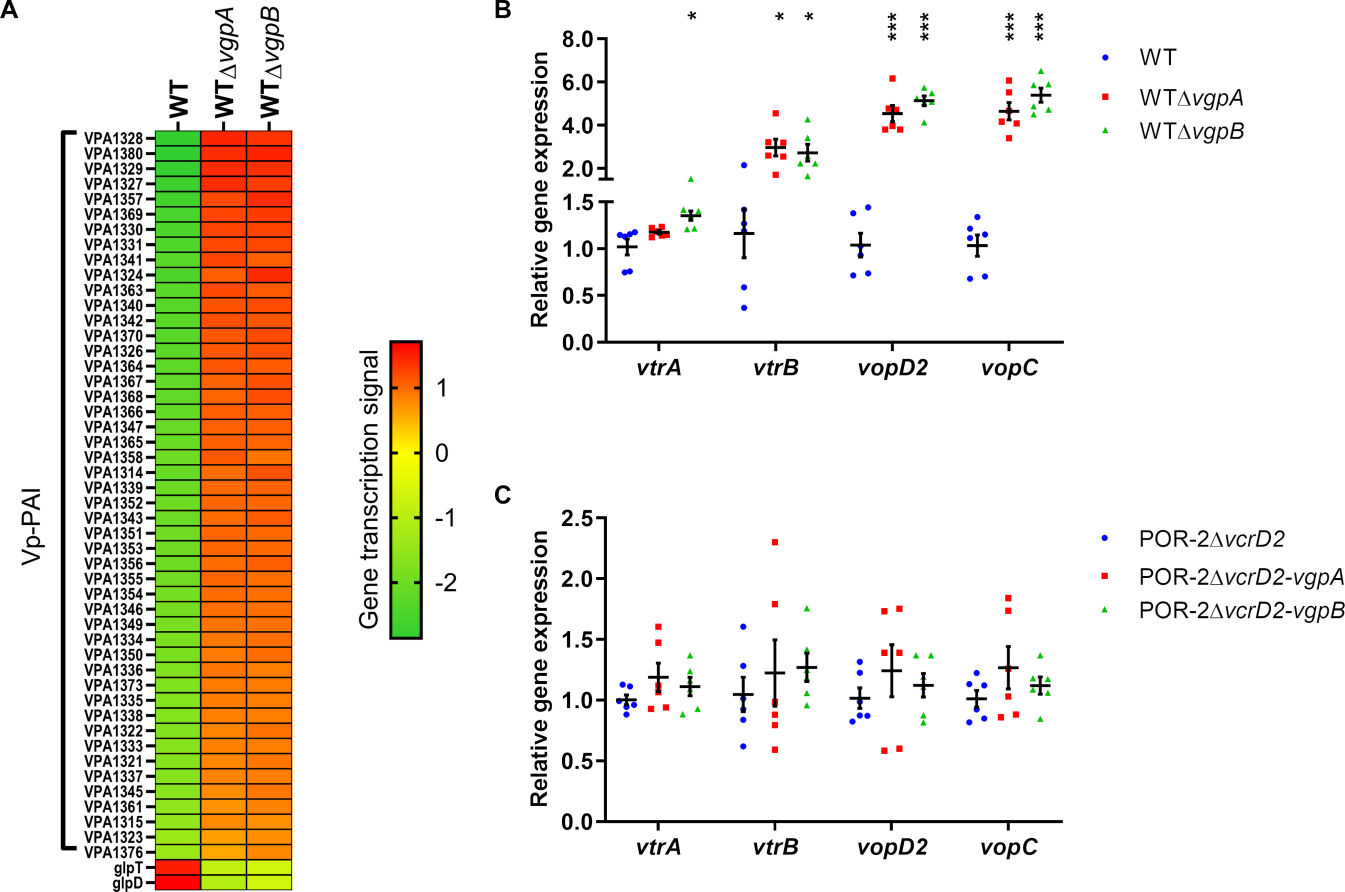
Deletions of the gatekeeper gene of T3SS2 lead to the upregulation of the Vp-PAI gene. (A) Gene transcription data from RNA-seq of *V. parahaemolyticus* RIMD2210633 (WT) and gatekeeper mutants (WT*∆vgpA* and WT*∆vgpB*); genes that showed more than 4-fold alteration with statistically significant differences (*P* ≤ 0.05) are shown. (B, C) Relative gene expression of *V. parahaemolyticus* WT and POR-2 (WT∆*tdhAS*∆*vcrD1*)∆*vcrD*2 with gatekeeper gene deletion was determined via qRT‒PCR using *recA* expression as an endogenous control and the expression of target genes by the parental strain as a baseline. The bars represent the average of three independent experiments. The error bars indicate 95% CI. ***, *P* ≤ 0.0001; **, *P* ≤ 0.001; *, *P* ≤ 0.05.

The gatekeeper (*vgpA* and *vgpB*) mutants had dysregulation of T3SS2 secretion that promoted effector but suppressed translocon secretions. The secretion phenomenon of gatekeeper mutants was similar to the host-cell contact state that activates T3SS expression in *Yersinia* spp., *P. aeruginosa*, *S. flexneri,* and *Bordetella* spp. (10–16). We, therefore, determined the transcriptome of gatekeeper mutants. RNA-seq analysis of *vgpA* and *vgpB* mutants revealed at least 4-fold changes in the upregulation of 48 Vp-PAI genes and the downregulation of 2 glycerophospholipid metabolism genes (*glpD* and *glpT*) (Fig. 1A; Table S1). qRT-PCR confirmed T3SS2-related gene upregulation in the gatekeeper mutants (Fig. 1B). To focus on T3SS2 function, excluding the cytotoxic effects of TDH and T3SS1, the POR-2 strain (WTΔ*tdhAS*Δ*vcrD1*), which is deficient in TDH and the T3SS1 export apparatus, was used as the parental strain for subsequent experiments. As gatekeeper mutants mimic host-cell contact states that switch substrate secretion from translocators to effectors, we determined the effect of T3SS2 secretion on T3SS2 expression. POR-2∆*vcrD2* (T3SS2 secretion defective background) was analyzed for T3SS2 gene expression. Subsequent analysis demonstrated that no T3SS2-related gene transcriptional changes in gatekeeper deletion in a T3SS2-secretion deficient background (Fig. 1C). The loss of T3SS2 secreted proteins from the bacterial cytosol facilitates the Vp-PAI gene transcription, indicating that a member of T3SS2 secreted proteins may play a negative transcriptional regulator of Vp-PAI.

### Identification of an unrecognized T3SS2-secreted protein that facilitates T3SS2-mediated infection and effector translocation

To identify an unrecognized T3SS2-secreted protein that alters the Vp-PAI expression, we analyzed the culture supernatant of gatekeeper mutants using LC-MS/MS. We generated *vgpA* and *vgpB* mutants in POR2*∆vopB2∆vopD2*, which lacked identified secreted proteins, including T3SS1-related proteins, TDH, VopB2, and VopD2, to discover unidentified and low-abundance T3SS2-secreted proteins. We compared the secreted proteins with those of the T3SS2-deficient strain (Fig. S1A and B). Proteomic analysis of the *V. parahaemolyticu*s secreted proteins revealed 109 proteins (Table S2). Among these proteins, 92 were detected in the supernatant of POR-2*∆vcrD2* (T3SS2 secretion defective strain)*,* which were classified as T3SS2-independent secreted proteins and excluded from further analysis. The remaining 17 proteins were considered T3SS2-secreted proteins. Among these, 13 were homologs of known T3SS proteins involved in *V. parahaemolyticus* pathogenicity (Fig. S1C). The other four candidate proteins are VPA1312, VPA1323, VPA1326, and VPA1369, encoded by Vp-PAI; however, their functions have not been reported.

**Fig 2:**
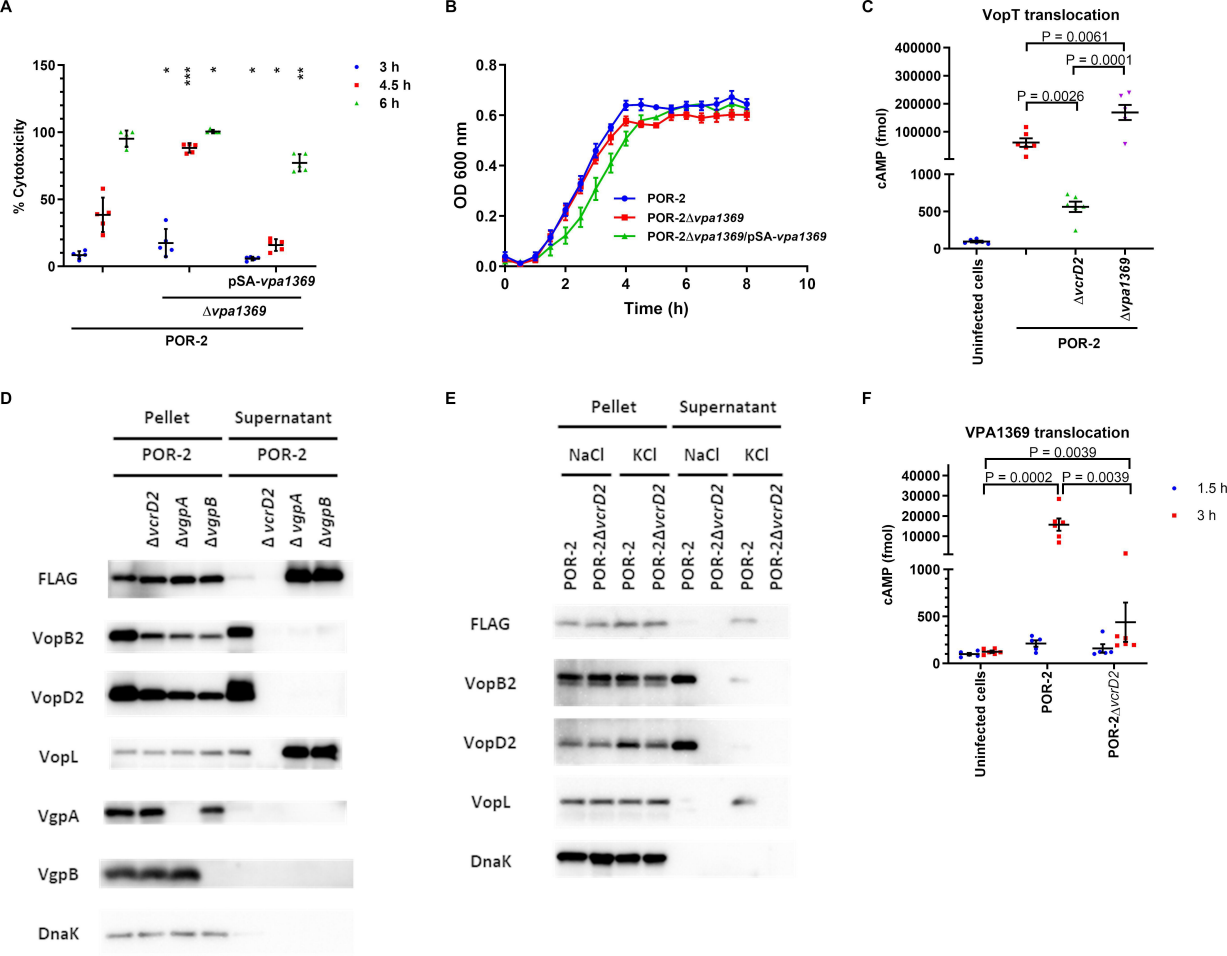
Identification of an unrecognized T3SS2-secreted protein that facilitates T3SS2-mediated infection and effector translocation. (A) Cytotoxic effect of *V. parahaemolyticus* POR-2 (WT∆*tdhAS*∆*vcrD1*) and the derivative strains on Caco-2 cells, with total cell lysis used as a positive control and uninfected cells used as a negative control. The bars represent the average of three independent experiments. The error bars indicate the standard deviations (SDs). ***, *P* ≤ 0.0001; **, *P* ≤ 0.001; *, *P* ≤ 0.05. (B) Growth curves of *V. parahaemolyticus* POR-2 and its derivative strains cultured in LB broth. The plots represent the average of three independent experiments. The error bars indicate the standard error of the mean (SEM). (C) Intracellular cAMP levels from VopT translocation in Caco-2 cells infected with VopT-CyaA-expressing isogenic *V. parahaemolyticus* POR-2, POR-2Δ*vcrD2* and POR-2Δ*vpa1369* for 1.5 h. The bars represent the average of three independent experiments. The error bars indicate the SEM. (D) Production and secretion of VPA1369 with a triple FLAG tag from *V. parahaemolyticus* POR-2 and derivative strains cultured in LB broth with 0.04% crude bile. DnaK was used as a control for sample preparation. The figure was representative of three independent experiments. (E) Production and secretion of VPA1369 with a triple FLAG tag from *V. parahaemolyticus* POR-2 and POR-2Δ*vcrD2* cultured in LB broth with 0.1 M NaCl or 0.1 M KCl. DnaK was used as a control for sample preparation. The figure was representative of three independent experiments. (F) Intracellular cAMP levels resulting from the translocation of VPA1369 in Caco-2 cells infected with VPA1369-CyaA-expressing isogenic *V. parahaemolyticus* POR-2 and POR-2Δ*vcrD2* for 1.5 or 3 h. The bars represent the average of three independent experiments. The error bars indicate the SEM.

Strains deficient in each gene were constructed using POR-2 as the parental strain to assess their cytotoxicity against Caco-2 cells, which serves as an indicator of T3SS2-dependent cytotoxicity (32). Deletion of *vpa1312*, *vpa1323*, or *vpa1326* had T3SS2-dependent cytotoxicity similar to the parental strain (Fig. S2A through C). In contrast, the *vpa1369* mutant initially exhibited cytotoxicity 3 h after infection and extremely damaged the infected cells within 4.5 h compared with POR-2 (Fig. 2A; Fig. S2D). The complemented strain significantly reduced the cytotoxic effect (Fig. 2A). However, gene deletion or complementation of the *vpa1369* did not affect bacterial growth compared with the parental strain (Fig. 2B). Additionally, using a CyaA-based VopT translocation assay to measure effector injection into host cells, a significant increase in VopT translocation was observed due to *vpa1369* deletion in POR-2 (Fig. 2C). These results indicated that *vpa1369* deletion facilitates T3SS2-dependent biological activity.

We confirmed the T3SS2-dependent secretion of VPA1369 by tagging its C-terminus with FLAG in POR-2, POR-2∆*vcrD2*, POR-2∆*vgpA,* and POR-2∆*vgpB*; therefore, FLAG detection by western blotting represents VPA1369 (Fig. 2D and E; Fig. S3 and S4). VgpA and VgpB production in the bacterial pellet and VopL, VopB2, and VopD2 secretion confirmed phenotypes of gatekeeper mutants and T3SS2 secretion deficient strain (Fig. 2D; Fig. S3). The presence of DnaK demonstrated comparable sample preparation. Consistent with the results of proteomic analysis, VPA1369 secretion was increased in the gatekeeper mutants (Fig. 2D; Fig. S3), and VPA1369 exhibited a secretion pattern similar to that of other T3SS2 effectors, such as VopL (8).

**Fig 3:**
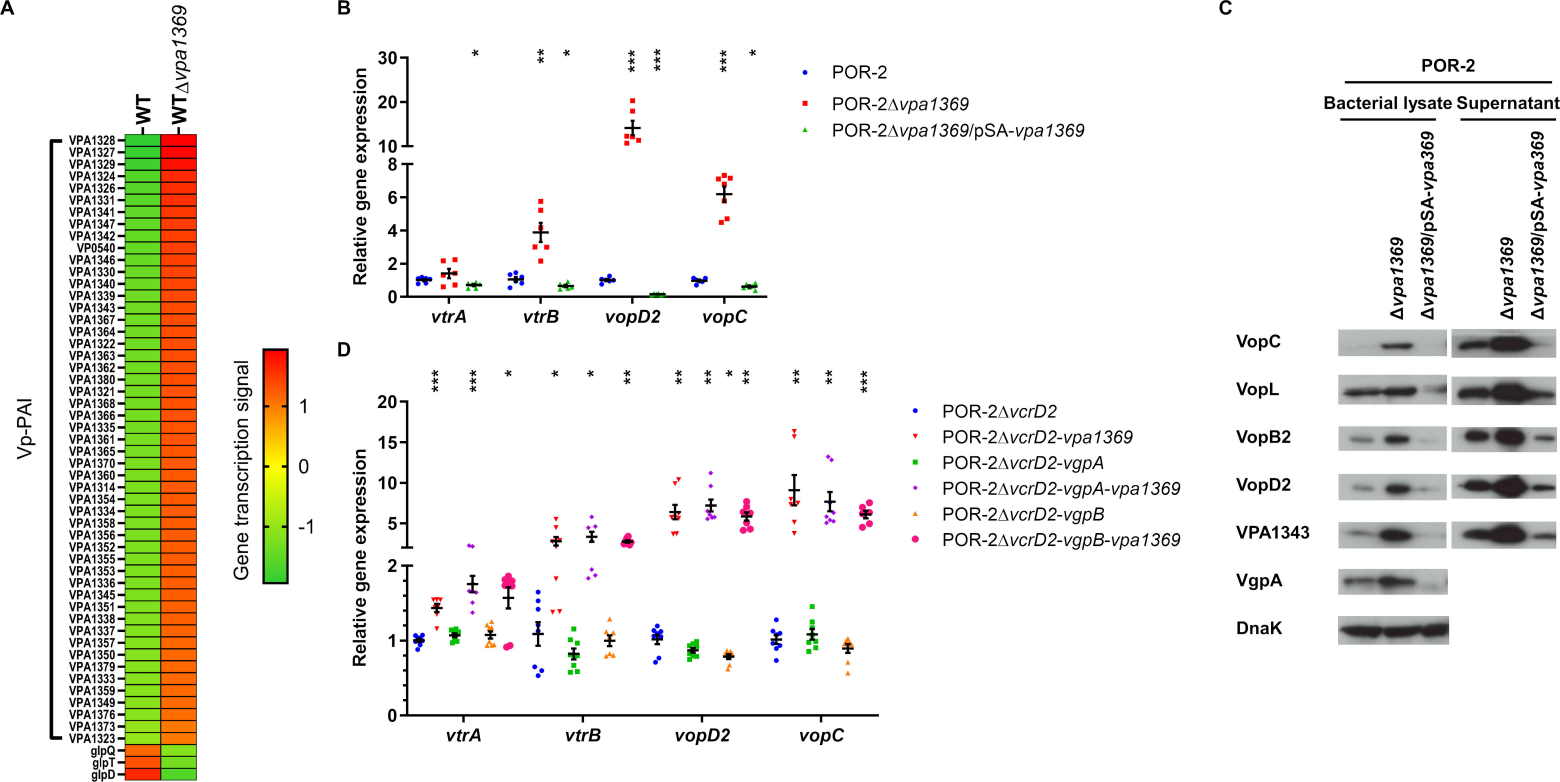
VPA1369 negatively regulates Vp-PAI-encoded gene expression. (A) Gene transcription levels were determined by RNA-seq of *V. parahaemolyticus* RIMD2210633 and the *vpa1369* mutant; genes that showed more than a 4-fold change in expression with a statistically significant difference (*P* ≤ 0.05) are shown. (B) Relative gene expression levels in the *V. parahaemolyticus* POR-2 (WT∆*tdhAS*∆*vcrD1*) with *vpa1369* deletion and complemented strain were determined via qRT-PCR, with *recA* expression as endogenous control and the expression of target genes by the parental strain as a baseline. The bars represent the average of three independent experiments. The error bars indicate 95% CI. ***, *P* ≤ 0.0001; **, *P* ≤ 0.001; *, *P* ≤ 0.05. (C) T3SS2-related protein production and secretion from *V. parahaemolyticus* POR-2 and its derivative strains cultured in LB broth. DnaK was used as a control for sample preparation. The figure was representative of three independent experiments. (D) Relative gene expression in the *V. parahaemolyticus* POR-2Δ*vcrD2* and derivative strains was determined via qRT-PCR, with *recA* expression as endogenous control and the expression of target genes by the parental strain as a baseline. The bars represent the average of three independent experiments. The error bars indicate 95% CI. ***, *P* ≤ 0.0001; **, *P* ≤ 0.001; *, *P* ≤ 0.05.

**Fig 4:**
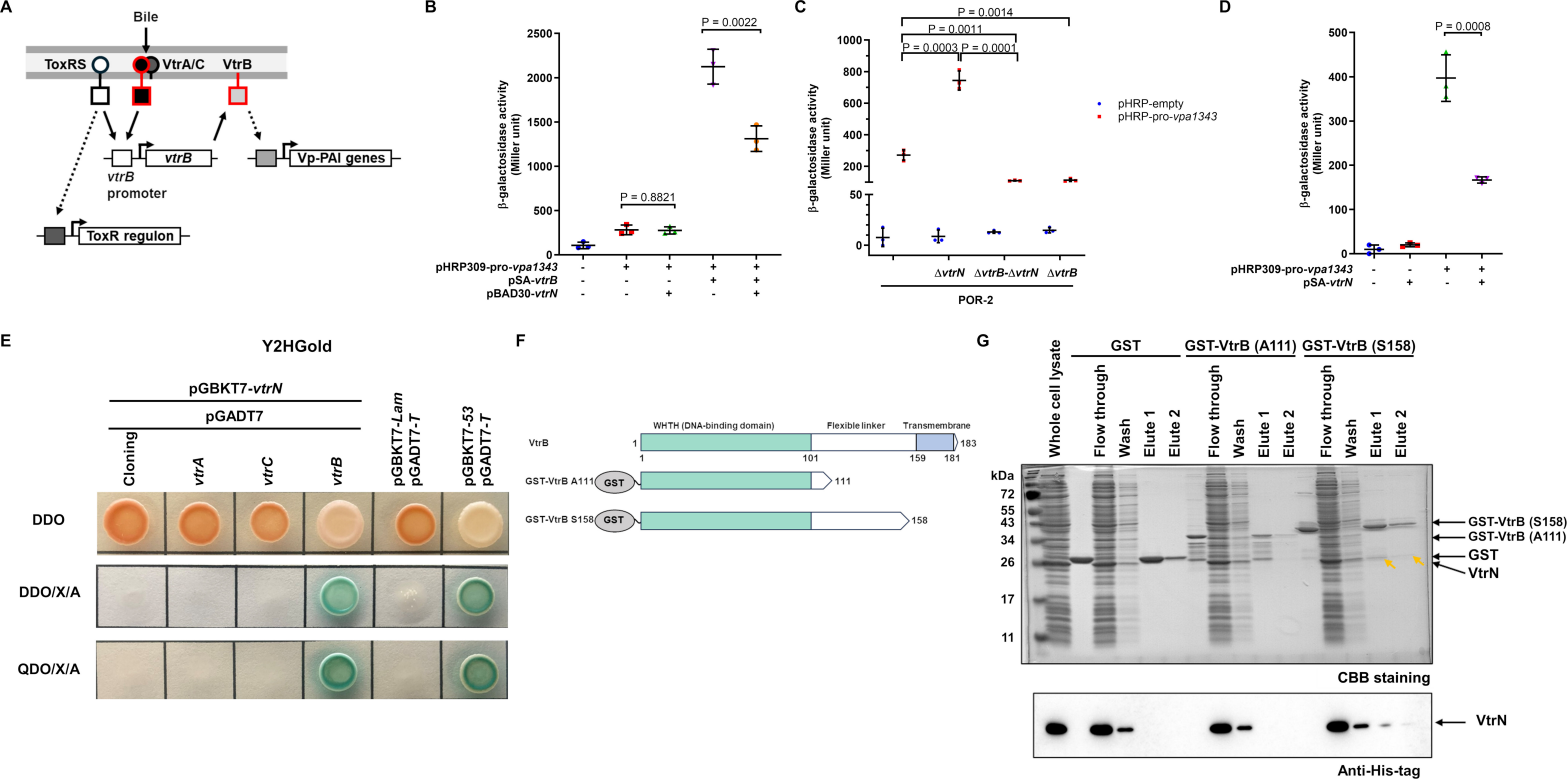
VtrN interacts with VtrB and suppresses VtrB-mediated transcriptional activity of Vp-PAI expression. (A) Diagram of the T3SS2 transcriptional regulatory system. (B) β-galactosidase activity resulting from the coexpression of VtrB (pSA-*vtrB*) and VtrN (pBAD30-*vtrN*) with a β-galactosidase reporter gene under the *vpa1343* promoter (pHRP309-pro-*vpa1343*) in *E. coli* MC4100 in LB broth supplemented with 0.02% arabinose. The bars represent the average of three independent experiments. The error bars indicate the SDs. (C) β-galactosidase activity of the β-galactosidase reporter gene under the *vpa1343* promoter (pHRP309-pro-*vpa1343*) in the *V. parahaemolyticus* POR-2 (WT∆*tdhAS*∆*vcrD1*) and derivative strains. The bars represent the average of three independent experiments. The error bars indicate the SDs. (D) β-galactosidase activity of the β-galactosidase reporter gene under the *vpa1343* promoter (pHRP309-pro-*vpa1343*) in *vtrN* mutant and complement strains cultured in LB broth. The bars represent the average of three independent experiments. The error bars indicate the SDs. (E) Protein-protein interactions between VtrN and other T3SS2 transcriptional regulators, Y2HGold/pGBKT7-*53* and pGADT7-*T* were used as positive controls. Co-transformants were cultured on a double dropout medium lacking leucine and tryptophan (SD/**‒**Trp/**‒**Leu; DDO; top panel), DDO supplemented with 40 µg/mL X-α-Gal and 200 ng/mL aureobasidin A (DDO/X/A; middle panel) and quadruple dropout media (SD/**‒**Ade/**‒**His/**‒**Leu/**‒**Trp; QDO) supplemented with 40 µg/mL X-α-Gal and 200 ng/mL aureobasidin A (QDO/X/A; bottom panel). Y2HGold/pGBKT7-*Lam* and pGADT7-*T* were used as negative controls. (F) Diagram of the VtrB domain and truncations of VtrB tagged with a GST at the N-terminus. (G) Coomassie brilliant blue staining and immunoblotting of VtrN with a hexahistidine tag pulled down from the bacterial lysate using GST-truncated VtrB. Yellow arrows indicated pull down VtrN.

We have previously reported that T3SS2 senses high K^+^ inside host cells, switching from secreting translocators to effectors. This switching can be reproduced without host cells by replacing NaCl with KCl in the culture medium (8). Accordingly, we examined VPA1369 secretion in the presence of Na^+^ or K^+^ (Fig. 2E; Fig. S4). The detections of translocator (VopB2 and VopD2) secretions in the presence of NaCl and effector (VopL) secretion in the presence of KCl confirmed the secretion pattern when *V. parahaemolyticus* was exposed to Na^+^ or K^+^ (Fig. 2E; Fig. S4). VPA1369 secretion by POR-2 was observed in the presence of KCl, mimicking host-cell contact, similar to VopL secretion (Fig. 2E; Fig. S4). Additionally, VPA1369 was not detected in the culture supernatant of the T3SS2-deficient strain, suggesting that T3SS2 specifically enhances VPA1369 secretion in the presence of KCl (Fig. 2E).

Given that the secretion behavior of VPA1369 resembled that of T3SS2 effectors, we investigated whether VPA1369 was translocated into host cells using a CyaA-based VPA1369 translocation assay (Fig. 2F). We observed a significant increase in intracellular cAMP levels in cells infected with POR-2 at 3 h post-infection compared with those in uninfected cells. In contrast, cAMP levels in cells infected with POR-2∆*vcrD2* were significantly lower than those in POR-2-infected cells, indicating that VPA1369 translocation was T3SS2 dependent. These results suggested that VPA1369 might have an effector role in host cells, which required further investigation.

### VPA1369 negatively regulates Vp-PAI-encoded gene expression

Bile acids, a potent activator of T3SS2 gene expression (27), influence the production of VPA1369 (Fig. S5A) and other T3SS2 components, possibly masking the VPA1369 function. We performed experiments without bile acids to examine the VPA1369 role in the free-living state. Because *vpa1369* deletion promoted T3SS2-dependent biological activity (Fig. 2A and C), RNA-seq was used to investigate VPA1369 in regulating Vp-PAI expression. RNA-seq detected the transcription of 4,587 genes (Table S3), among which 54 genes showed significant changes at least 4-fold due to *vpa1369* deletion: 51 genes were upregulated, and three genes were downregulated (*P* ≤ 0.05; Fig. 3A). All genes with significantly altered expression were encoded by Vp-PAI, except for *vp0540* (carbon starvation protein A) and three *glp* family genes (*glpD*, *glpQ*, and *glpT*). The expression patterns of these genes in the *vpa1369*-deficient strain were similar to gatekeeper mutants (Fig. 1A). POR-2 strain (WTΔ*tdhAS*Δ*vcrD1*), which is deficient in TDH and the T3SS1 export apparatus, was used as the parental strain for subsequent experiments to focus on T3SS2 and *vpa1369* functions and avoid the cytotoxic effects of TDH and T3SS1. qRT-PCR confirmed the increased Vp-PAI gene transcription in the POR-2Δ*vpa1369* (Fig. 3B). This increased transcription was reversed by complementation. Immunoblotting confirmed the gene expression results, showing that the production of T3SS2-related proteins was increased in the *vpa1369* mutant but decreased in the complemented strain (Fig. 3C). However, unlike gatekeeper mutations that alter types of substrate secretion (Fig. 2D), the *vpa1369* deletion caused the increased production and secretion of all kinds of T3SS2 secretion substrates (Fig. 3C; Fig. S5B through F). Although increased Vp-PAI gene transcription by gatekeeper mutants was dependent on T3SS2 secretion (Fig. 1B and C), T3SS2 secretion did not influence Vp-PAI upregulation in the *vpa1369* mutant (Fig. 3D). These findings indicated that VPA1369 directly represses the Vp-PAI expression as a negative regulator. Thus, we named VPA1369 *V. parahaemolyticus*-negative transcriptional regulator (VtrN).

### VtrN interacts with VtrB and suppresses VtrB-mediated transcriptional activity for Vp-PAI expression

We examined how VtrN negatively regulates Vp-PAI gene transcription. VtrN was predicted to contain DUF3528 (of an unidentified protein family) at amino acid positions 139-183 via a MOTIF search (https://www.genome.jp/tools/motif/). However, VtrN did not show homology to known proteins or possess a DNA-binding domain. These findings suggest that VtrN may affect the gene expression cascade of Vp-PAI rather than directly binding to the regulatory region of Vp-PAI genes. As shown in Fig. 4A, the VtrAC complex positively regulates the Vp-PAI expression, which cooperates with ToxRS to transcribe T3SS2 master transcriptional regulator VtrB (21, 22, 26, 27, 33).

The transcriptional profiles of the gatekeeper or *vtrN* mutants revealed increased expression of *vtrA*, *vtrB*, and *vtrC* but did not alter *toxRS* expressions (Fig. S6A, Tables S1 and S3). ToxR transcriptional activity was also monitored by a reporter assay in *V. parahaemolyticus* POR-2. The promoter of *ompU*, one of the ToxR regulons, was constructed to control the expression of β-galactosidase (Fig. S6B). No change in transcriptional activation was observed with the deletion of either *vtrB* or *vtrN*. Additionally, activation of the *vtrB* promoter by the induction of VtrA could be reproduced by the β-galactosidase reporter gene assay in *Escherichia coli*, whereas VtrN expression did not affect this VtrA-mediated activation of the *vtrB* promoter (Fig. S6C). In contrast, in a reporter assay conducted in the *E. coli* system to monitor VtrB transcriptional activity via the *vpa1343* promoter (one of the operons within Vp-PAI), VtrB-induced activation of the *vpa1343* promoter was reduced by 38% upon co-expression of *vtrN* (Fig. 4B). This VtrN inhibitory effect on the VtrB transcriptional activity was confirmed in *V. parahaemolyticus* (Fig. 4C). *vpa1343* promoter activity was increased by *vtrN* deletion. This increase was reversed by double deleting *vtrB* and *vtrN* genes, achieving the same level as in *vtrB* single mutant. Additionally, complementation with the *vtrN* gene reduced β-galactosidase activity (Fig. 4D). These findings suggest that VtrN specifically represses the VtrB transcriptional activity but does not affect VtrAC or ToxRS in Vp-PAI expression.

Having determined that VtrN represses VtrB transcriptional activity, we investigated protein-protein interactions between VtrN and the transcriptional regulators VtrA, VtrB, and VtrC via a yeast two-hybrid system (Fig. 4E). Co-transformants harboring pGBKT7-*vtrN* and pGADT7-*vtrA*, pGADT7-*vtrB*, or pGADT7-*vtrC* were grown on DDO agar, but only co-transformants harboring pGBKT7-*vtrN* and pGADT7-*vtrB* were able to develop blue colonies on DDO/X/A, as well as a positive control. Similar results on a stringent selection plate QDO/X/A indicated a tight and specific interaction between VtrN and VtrB.

Pull-down assay further confirmed the interaction between VtrN and VtrB. VtrB is the transmembrane transcriptional activator of T3SS2, with a winged helix-turn-helix DNA-binding domain of the OmpR family in its N-terminal region (1-101 a.a.) and a transmembrane domain in its C-terminal region (159-181 a.a.) (Fig. 4F). The purified GST-fused helix-turn-helix domain of VtrB (GST-VtrB (A111)) and the truncated VtrB without a transmembrane region (GST-VtrB (S158)) were coated on glutathione beads to capture VtrN with an N-terminal hexahistidine tag (His_6_-VtrN) in the bacterial lysate (Fig. 4F and G). His_6_-VtrN was detected in the elution fractions only when glutathione beads were coated with GST-VtrB (S158), indicating that VtrN interacted with the cytoplasmic region of VtrB, specifically requiring its flexible linker domain.

## DISCUSSION

**Fig 5:**
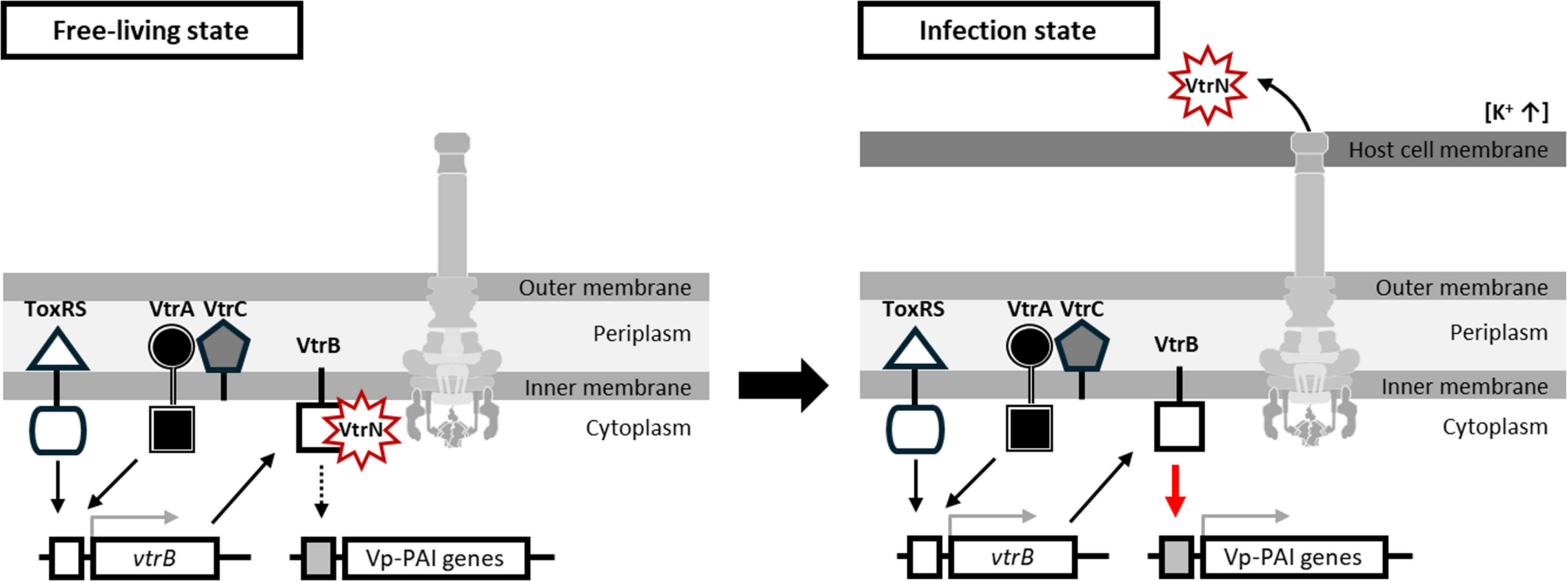
Schematic describing the role of VtrN in Vp-PAI regulation. VtrN directly binds VtrB to block the transcription activity during the free-living state. Upon host-cell contact, bacteria sense intracellular K^+^, and VtrN is translocated into the host cytosol, activating VtrB transcriptional activity of Vp-PAI.

T3SS is a complex and highly specialized apparatus that injects effector proteins directly into host cells. Recent advances in genomic technology have shown that T3SS exists in hundreds of bacterial species (1, 34). Host-cell contact-dependent regulation of gene expression is significant in the infection. However, it is largely unknown for most T3SSs, except well-studied T3SSs such as those of *P. aeruginosa* and *Yersinia* spp. This study revealed a previously unknown mechanism for the host-cell contact-dependent induction of T3SS2 expression in *V. parahaemolyticus. V. parahaemolyticus* T3SS can be regulated by the ToxRS system that functions in a cell density-dependent manner (24–26). However, VtrN negatively regulates Vp-PAI expression during the free-living state by suppressing the VtrB transcription activity until the bacteria achieve host-cell contact. The negative feedback control by repressing Vp-PAI expression of VtrN during the free-living state may be advantageous for optimizing its growth and maintaining stability. Sensing intracellular K^+^ leads to VtrN secretion, which impedes the negative feedback loop and activates VtrB transcriptional activity. Therefore, Vp-PAI expression upregulates to promote bacterial virulence during infection (Fig. 5).

The T3SSs, which are evolutionarily linked to the flagellum, share structural, functional, and regulatory similarities (4, 35). These systems coordinate gene expression with secretory activity (36). In *Salmonella enterica* serovar Typhimurium and *E. coli*, flagellar gene expression is coupled with assembly (37). Conversely, T3SS injectosomes are tightly regulated in response to environmental signals, especially host-cell contact (4). Each injectosome responds differently to environmental stimuli to upregulate T3SS expression (36). The T3SSs of *P. aeruginosa* and *Yersinia* spp. sense low Ca^2+^ (5, 6), whereas *V. parahaemolyticus* T3SS2 responds to high K^+^ from host cells (8). These stimuli trigger changes in secreted substrates, protein secretion profiles, and regulatory molecule interactions within bacterial cells. Despite having diverse signaling pathways, the signals eventually converge upon activation of the T3SS master regulator (4). These findings underscore the importance of host-cell contact-dependent mechanisms in initiating T3SS expression during infection.

In *P. aeruginosa,* the injectosome utilizes ExsA to activate T3SS expression. Regulation occurs via a cascade involving ExsD, ExsC, and ExsE. Under non-inducing conditions, ExsD binds to and inhibits ExsA. ExsE, a secreted protein, binds to ExsC and prevents its interaction with ExsD. ExsE is exported from the cell when secretion is active, allowing ExsC to inhibit ExsD and freeing ExsA to initiate T3SS expression (10, 11). The ExsACDE regulatory cascade also regulates *V. parahaemolyticus* T3SS1 expression (38, 39). The VtrN activity in *V. parahaemolyticus* T3SS2 is similar to that of ExsD in that VtrN binds to the master regulator VtrB as an anti-activator and represses VtrB transcriptional activity (Fig. 4). However, unlike the Exs system, VtrN is a T3SS2-secreted substrate. Moreover, the Vp-PAI region lacks proteins homologous to Exs proteins. Thus, T3SS2 may not require multiple signaling cascades, as in the Exs system, as VtrN, a VtrB anti-activator, is directly secreted from T3SS2 under the control of gatekeepers.

The *Yersinia* spp. injectosome is expressed under low calcium levels and upon host-cell contact, as observed in the *P. aeruginosa* T3SS (6). The negative regulation of T3SS expression under non-inducing conditions is carried out by a regulatory complex comprising three proteins: YopD, LcrH, and LcrQ (40). This negative regulator complex binds to transcripts of the master regulators *lcrF* and Ysc-Yop T3SS, which may prevent ribosome binding and accelerate mRNA degradation (12, 41–44). This study revealed that VtrN interacts with VtrB to suppress its transcriptional activity (Fig. 4), suggesting a possible mechanism of VtrB repression via the protein-protein interactions. However, the possible involvement of VtrN in post-transcriptional regulation requires further investigation.

The *bsc*-encoded genes of the *Bordetella* injectosome are highly conserved among *B. pertussis*, *B. parapertussis*, and *B. bronchiseptica* (45). *Bordetella* T3SS expression is regulated by BtrS (46). BtrA/BspR has been identified as a T3SS-secreted BtrS antagonist (15, 16), but whether BtrA/BspR binds directly to the master regulator BtrS and inhibits its transcriptional activity is controversial (15, 16). Future comparative studies are needed to analyze and understand the mechanisms of action of VtrN on VtrB and BtrA/BspR on BtrS in more detail.

In *Shigella* injectosomes, host-cell contact activates secretion and T3SS effector gene transcription, which MxiE regulates (14). IpgC enhances MxiE activity, whereas OspD1, through interaction with Spa15, a co-anti-activator chaperone, inhibits MxiE under non-inducing conditions (47). Upon induction, OspD1, IpaB, and IpaC are secreted, MxiE is released, and IpgC binds to MxiE to activate transcription (13). Thus, the mode of action of OspD1 as an anti-activator is similar to that of VtrN; however, the inhibitory effect of OspD1 on T3SS gene expression is limited at the protein level to one effector protein, IpaH. In addition, various chaperone interventions have also been reported as regulators of contact-dependent gene regulation in other injectosomes (e.g., ExsC in *P. aeruginosa*, SycH against LcrQ and LcrH against YopD in *Yersinia* spp., Spa15 against OspD1 and IpgC against IpaB/C in *Shigella* spp.). In the present study, chaperones involved in contact-dependent regulation of T3SS2 gene expression were not identified. The fact that gene regulation by VtrN is associated with the secretory activity of T3SS2 suggests that unidentified chaperone(s) may be involved in the secretion of VtrN or the binding and activation of the master regulator VtrB.

The unique mechanisms and signaling pathways involved in the host-cell contact-dependent regulation of T3SS2 highlight its distinctiveness compared with other injectosomes. Although no regulatory molecules such as VtrN have been found in other T3SSs, host-cell contact still influences the active state or expression of the T3SS master regulator through anti-activators, suggesting functional conservation across T3SSs. Tight control of T3SS gene expression balances virulence with bacterial survival and growth, which is crucial for pathogenic bacteria *in vivo* (48). This is because rapid changes in gene expression levels are known to regulate T3SS output and optimize growth by creating trade-offs between virulence costs and the metabolic capacity of the pathogen (49). Understanding these regulatory mechanisms could lead to innovative infection control strategies, opening new avenues for research and discovery.

## MATERIALS AND METHODS

### Bacterial and yeast strains and culture conditions

*V. parahaemolyticus* RIMD2210633 (WT) and POR-2 (WTΔ*tdhAS*Δ*vcrD1*; TDH and T3SS1 export apparatus deficient strain) were obtained from the Pathogenic Microbes Repository Unit, International Research Center for Infectious Diseases, Research Institute for Microbial Diseases, Osaka University, Japan. *E. coli* DH5α and SM10λ*pir* were used for mutagenesis. *E. coli* MC4100 was used for the β-galactosidase reporter gene assay. *E. coli* BL21(DE3) was used for the pull-down assay. Bacteria were grown in LB broth containing 0.5% NaCl, 0.1 M NaCl, or 0.1 M KCl at 37°C. *Saccharomyces cerevisiae* Y2HGold (Clontech) was used to determine protein-protein interactions. *S. cerevisiae* was grown on YPDA agar at 30°C for 3–5 days. The strains and plasmids generated and used in this study are listed in Tables S4 and S5.

### Mutagenesis

Mutagenesis was performed via the allelic replacement method as described by Kodama et al. (32). In brief, a fragment of 332 bp deleted *vtrN* was cloned into a pYAK1 containing the *sacB* gene. The deletion fragment in pYAK1 was transformed into *V. parahaemolyticus*. SacB-based sucrose counterselection was performed to induce allelic replacement. The same procedure was used to generate a triple-FLAG tag at the C-terminus of VtrN. The complete *vtrN* sequence was cloned into pSA-*tdh*P (32) and then transformed into the *vtrN* mutant for complementation. The plasmids and sequences of primers used for mutagenesis and complementation are shown in Tables S5 and S6.

### RNA extraction

Thirty microliters of overnight culture of *V. parahaemolyticus* was inoculated in 3 mL of LB broth and then incubated at 37°C for 3 h. The bacterial culture was treated with RNAprotect Bacteria Reagent (Qiagen), and RNA was extracted via RNeasy Mini Kit (Qiagen). Contaminated genomic DNA was eliminated via the RNase-Free DNase Set (Qiagen). DNA contamination was verified by amplifying the *recA* gene, in which no samples produced PCR products.

### RNA-seq

Thirty microliters of overnight culture of *V. parahaemolyticus* was inoculated in 3 mL of LB broth and then incubated at 37°C for 3 h. Total RNA was stabilized via RNAprotect Bacteria Reagent (Qiagen) and extracted via RNeasy kit (Qiagen). Ribosomal RNA was removed, and the remaining RNA was fragmented via the Ribo-Zero Plus rRNA Depletion Kit (Illumina). The RNA libraries were prepared via the TruSeq stranded mRNA Library Prep Kit (Illumina) without purification or fragmentation. Sequencing was performed on a HiSeq 3000 platform in 75-base single-end mode. The generated reads were mapped to the *V. parahaemolyticus* RIMD2210633 reference genome (GCF_000196095.1_ASM19609v1_genomic.fna) via TopHat v2.1.1 in combination with Bowtie2 ver.2.2.8 and SAMtools ver.0.1.18. Fragments per kilobase of exon per million mapped fragments (FPKMs) were calculated via Cuffnorm 2.2.1. Statistical analysis was performed with Subio software (https://www.subioplatform.com/, Subio Inc.). Genes with FPKMs ≥ 0.1 were included in the analysis.

### qRT-PCR

T3SS2 gene expression was determined via RNA-direct SYBR Green real-time PCR master mix (Toyobo, Japan). The *recA* expression was used to normalize the expression of the target genes. The normalized target gene expression levels of the mutant or complemented strains were compared with those of the parental strain. The relative gene expression was calculated via the 2^-∆∆Ct^ method. The sequences of primers used for gene expression are shown in Table S6.

### *V. parahaemolyticus* lysate and cell-free culture supernatant preparation

*V. parahaemolyticus* strains were cultured in LB broth supplemented with 0.5% (wt/vol) NaCl, 0.1 M NaCl, or 0.1 M KCl at 37°C for 6 h. The bacteria were collected from 1 mL of culture. The proteins in the cell-free culture supernatant were precipitated with trichloroacetic acid at a final concentration of 14% (vol/vol). The bacteria and protein pellets were resuspended in Laemmli buffer and heated at 95°C for 10 min for immunoblotting.

### LC-MS/MS

The proteins from the cell-free culture supernatants were prepared as described above. The samples were purified via methanol‒chloroform precipitation and dissolved in 20 µL of 0.1% RapiGest (Waters). The protein solutions were reduced with 10 mM dithiothreitol and alkylated with 55 mM iodoacetamide. The resulting protein solutions were digested with mass spectrometry-grade trypsin (Promega) overnight at 37°C. The trypsinized protein solutions were analyzed via an LC-MS/MS system consisting of an HTC-PAL autosampler, Michrom nano-Advance UHPLC (Michrom BioResources Inc.), and an LTQ Orbitrap Velos mass spectrometer (Thermo Fisher Scientific). An ODS L-column (3 µm, 0.1 × 150 mm, CER) was employed for peptide separation, and another ODS L-column (5 µm, 0.3 × 5 mm, CERI) was used as the trap column. The mobile phase consisted of water containing 0.1% (vol/vol) formic acid and acetonitrile. The peptides were eluted via a linear gradient of 5-35% acetonitrile for 45 min at 500 nL/min. The LTQ Orbitrap Velos instrument was operated in data-dependent mode to switch between full-scan MS and MS/MS acquisition automatically. The mass spectrometric conditions were as follows: ion spray voltage, 1.9 kV; HCD collision energy, 35%; mass range, m/z 350-1500; and dynamic exclusion, 60 s.

The raw data were converted to MGF files via Proteome Discoverer 1.4 (Thermo Fisher Scientific) and searched against the NCBInr_20160401 database (selected for Proteobacteria, 2,956,7748 entries) via Mascot Server 2.5.1 (Matrix Science). The precursor mass tolerance was 10 ppm, and the MS/MS tolerance was 0.8 Da for the Orbitrap and linear ion trap, respectively. The carbamidomethyl of cysteine was specified in Mascot as a fixed modification. Gln->pyro Glu at the N-terminus, oxidation of methionine, and acetylation of the N-terminus were specified in Mascot as variable modifications. A peptide significance threshold of *P* < 0.05 was used for post-search filtering. The quantitative values and fold changes were calculated via Scaffold 3.4.5 (Proteome Software) for MS/MS-based proteomic studies (50).

### Immunoblot

Proteins were separated by SDS-PAGE, transferred to a PVDF membrane, and probed with the serum of rabbits immunized against VopC, VopL, VopB2, VopD2, or VPA1343. The samples were then probed with a horseradish peroxidase-conjugated goat anti-rabbit antibody. The blots were developed via an ECL Western blotting Kit (GE Healthcare). In the pull-down assay, mouse monoclonal anti-6xhistidine antibody (9C11) (Fujifilm, Wako) and horseradish peroxidase-conjugated rabbit anti-mouse antibody (Invitrogen) were used.

### Cytotoxicity

Caco-2 cells (human colon epithelial cell line; HTB-37) were prepared and infected with *V. parahaemolyticus* at MOI of 100, as described previously (8). Cytotoxicity was investigated by measuring the lactate dehydrogenase in the culture supernatant using a CytoTox96 Non-Radioactive Cytotoxicity Assay (Promega).

### CyaA-based translocation assay

*V. parahaemolyticus* cells harboring plasmids expressing VopT or VtrN fused to the catalytic domain of CyaA at the C-terminus were cocultured with Caco-2 cells at MOI of 100 in duplicate wells for 1.5 h or 3 h. The translocation of VopT or VtrN conjugated with CyaA converted intracellular AMP to cAMP by intracellular calmodulin-activated CyaA activity. The intracellular cAMP levels in infected cells were determined via a cAMP Biotrak Enzyme immunoassay system (GE Healthcare) according to the manufacturer’s instructions.

### β-Galactosidase reporter gene assay

The expression of the β-galactosidase reporter gene in the pHRP309 plasmid was controlled by *vtrB* or *vpa1343* promoter. Reporter gene transcription was activated by *vtrA* or *vtrB* expression from the pSA vector. Arabinose induction activated *vtrN* expression from pBAD30. An empty vector was used instead of the desired gene or promoter for the controls. Thirty microliters of overnight culture of *E. coli* and *V. parahaemolyticus* with a reporter gene and associated plasmids were inoculated in 3 mL of LB broth containing 0.02% arabinose and then incubated at 37°C for 6 h before measuring β-galactosidase activity. β-galactosidase activity was determined in cell lysates via Miller’s method (51) with *o-*nitrophenyl-β-D-galactopyranoside as a substrate.

### Protein-protein interaction analysis via the yeast two-hybrid system

Full-length *vtrA*, *vtrC*, *vtrB*, and *vtrN* genes were cloned into pGBKT7 or pGADT7 (Clontech) for the expression of a protein that was fused to the GAL4 DNA-binding domain or GAL4 activator domain. The sequences of primers used are shown in Table S6. The target gene was ligated into pGBKT7 and pGADT7 between EcoRI and BamHI restriction enzyme restriction sites. Each purified plasmid was introduced into *S. cerevisiae* strain Y2HGold (Clontech) using the yeast transformation system 2 (Clontech). Single dropout medium lacking tryptophan or leucine was used to select Y2HGold strains harboring pGBKT7 or pGADT7. Autoactivation was performed by subculturing on an agar plate with a single dropout medium containing 40 µg/mL X-α-Gal (SD/-Trp/X-α-Gal for Y2HGold with pGBKT7 vector and SD/-Leu/X-α-Gal agar plate for Y2HGold with pGADT7 vector). None of the clones exhibited autoactivation.

A double transformation was performed by delivering pGBKT7 and pGADT7 into *S. cerevisiae* to investigate protein-protein interactions. A double dropout medium lacking leucine and tryptophan (DDO; SD/-Trp/-Leu) was used to select the transformed *S. cerevisiae* strain Y2HGold carrying the pGADT7 and pGBKT7 vectors. Protein-protein interactions were determined by observing growth of *S. cerevisiae* with a double transformation of pGBKT7 and pGADT7 on a double dropout medium lacking leucine and tryptophan (DDO; SD/**‒**Trp/**‒**Leu) and quadruple dropout media (QDO; SD/**‒**Ade/**‒**His/**‒**Leu/**‒**Trp) supplemented with 40 µg/mL X-α-Gal and 200 ng/mL aureobasidin A (DDO/X/A and QDO/X/A). Y2HGold with pGBKT7-*vtrN* and pGADT7-gene of interest was subcultured on DDO medium and incubated at 30°C for 3 days. A single yeast colony was suspended in 50 µL of distilled water. Ten microliters of the suspension were dropped onto DDO/X/A and QDO/X/A and dried. The plates were incubated at 30°C for 4 days. Y2HGold with pGBKT-*53* and pGADT7-*T* was used as a positive control. Y2HGold with pGBKT7-*Lam* and pGADT7-*T* was used as a negative control.

### Pull-down assay

GST-fused VtrB (GST-VtrB) was expressed from the plasmid pGEX6P1-*vpa1348* (A111) or (S158) in *E. coli*. Hexahistidine-tag-fused VtrN (His_6_-VtrN) was expressed from the plasmid pCold1-*vpa1369*. For the pull-down assay, 500 µg of GST-VtrB was applied to a glutathione‒Sepharose column, and a cell suspension containing His_6_-VtrN was applied to the column. The column was washed with 10-column volumes of D-PBS(-) solution, and GST-VtrB and its binding proteins were eluted with TN buffer (20 mM Tris-HCl pH 8.0, 100 mM NaCl) supplemented with 15 mM reduced glutathione.

### Statistical analysis

All graphs represent the mean values from at least three independent experiments. The distribution of the data in each group was determined using the Shapiro-Wilk W test. A two-tailed Student’s *t*-test was used for the statistical analysis of data with normal distribution. Mann-Whitney test was performed for data that did not have a normal distribution. A *P* value (*P*) ≤ 0.05 was considered indicative of statistical significance.

## Data Availability

The transcriptomic data of *V. parahaemolyticus* in this study were deposited in the GEO database with accession no. GSE266863.

## SUPPLEMENTAL MATERIAL

Supplemental figures msystems.00251-25-s0001.pdf Fig. S1 to S6.

Supplemental captions msystems.00251-25-s0002.pdf Captions for Tables S1 to S6.

Supplemental tables msystems.00251-25-s0003.xlsx Tables S1 to S6.

ASM does not own the copyrights to Supplemental Material that may be linked to, or accessed through, an article. The authors have granted ASM a non-exclusive, world-wide license to publish the Supplemental Material files. Please contact the corresponding author directly for reuse.
