# Supplementary material for: Regulatory mechanism for host-cell contact-dependent T3SS gene expression in Vibrio parahaemolyticus
Source: mSystems. 2025 Jun 17;10(7):e00251-25. doi: 10.1128/msystems.00251-25 (PMC12282083; doi:10.1128/msystems.00251-25)
Supplement: Supplemental figures — Fig. S1 to S6. [file msystems.00251-25-s0001.pdf]

Supplementary figures

Regulatory mechanism for host-cell contact-dependent T3SS gene expression in *Vibrio parahaemolyticus*

Sarunporn Tandhavanant[1,2], Hiroyuki Terashima[1,3], Hirotaka Hiyoshi[1], Dhira Saraswati Anggramukti[4], Nopadol Precha[2,5], Tetsuya Iida[4], Shigeaki Matsuda[4,] Narisara Chantratita[2], Toshio Kodama[1*]

[1]Department of Bacteriology, Institute of Tropical Medicine, Nagasaki University, Nagasaki, Japan

[2]Department of Microbiology and Immunology, Faculty of Tropical Medicine, Mahidol University, Bangkok, Thailand

[3]Department of Pharmacology, College of Pharmacy, Kinjo Gakuin University, Nagoya, Japan

[4]Department of Bacterial Infections, Research Institute for Microbial Diseases, Osaka University, Osaka, Japan

[5]Department of Environmental Health and Technology, School of Public Health, Walailak University, Nakhon Si Thammarat, Thailand

***Address correspondence to** Toshio Kodama, tkodama@nagasaki-u.ac.jp

A

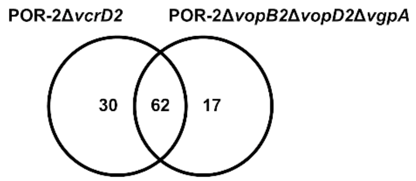

B

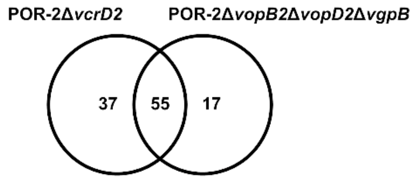

C

Proteins				Total spectrum count		
Name		Function	References	POR-2Δ*vcrD2*	POR-2Δ*vopB2*Δ*vopD2*Δ*vgpA*	POR-2Δ*vopB2*Δ*vopD2*Δ*vgpB*
VPA1370	(VopL)	T3SS2 effector	Liverman ADB, et al., 2007	0	105	97
VPA1357	(VopV)	T3SS2 effector	Okada N, et al., 2009; Hiyoshi H, et al., 2011	0	98	87
VPA1369		Hypothetical protien	-	0	28	39
VPA1321	(VopC)	T3SS2 effector	Kodama T, et al., 2007	0	19	11
VPA1329	(VopO)	T3SS2 effector	Hiyoshi H, et al., 2015	0	12	13
VPA1380	(OspB)	T3SS2 effector	Ono T, et al., 2006; Calder T, et al., 2014	0	23	14
VPA1326		Hypothetical protien	-	0	17	16
VPA1350		T3SS2 secretion/translocation apparatus	Zhou X, et al., 2013	0	5	5
VPA1346	(VopP)	T3SS2 effector	Park KS, et al., 2004	0	10	13
VPA1345	(VopW)	T3SS2 translocation apparatus	Zhou X, et al., 2012	0	3	3
VPA1327	(VopT)	T3SS2 effector	Kodama T, et al., 2007	0	9	7
VPA1328	(VopG)	T3SS2 effector	Plaza N, et al., 2021	0	6	5
VPA1312		Hypothetical protien	-	0	8	6
VPA1368		EscI/YscI/HrpB family type III secretion system inner rod protein	-	0	6	2
VPA1323		Hypothetical protien	-	0	2	2
VPA0460		PAS factor family proteins	Makino K, et al., 2003	0	2	2
VP0129		phosphoenolpyruvate carboxykinase	Makino K, et al., 2003	0	2	3

Fig. S1 Identification of T3SS2-secreted proteins influenced by loss of the gatekeeper component. Venn diagrams of the number of secreted proteins from the *vgpA* mutant (A) and *vgpB* mutant (B) compared with POR-2Δ*vcrD2*. (C) Total spectral count of 17 T3SS2-dependent secreted proteins upon gatekeeper loss.

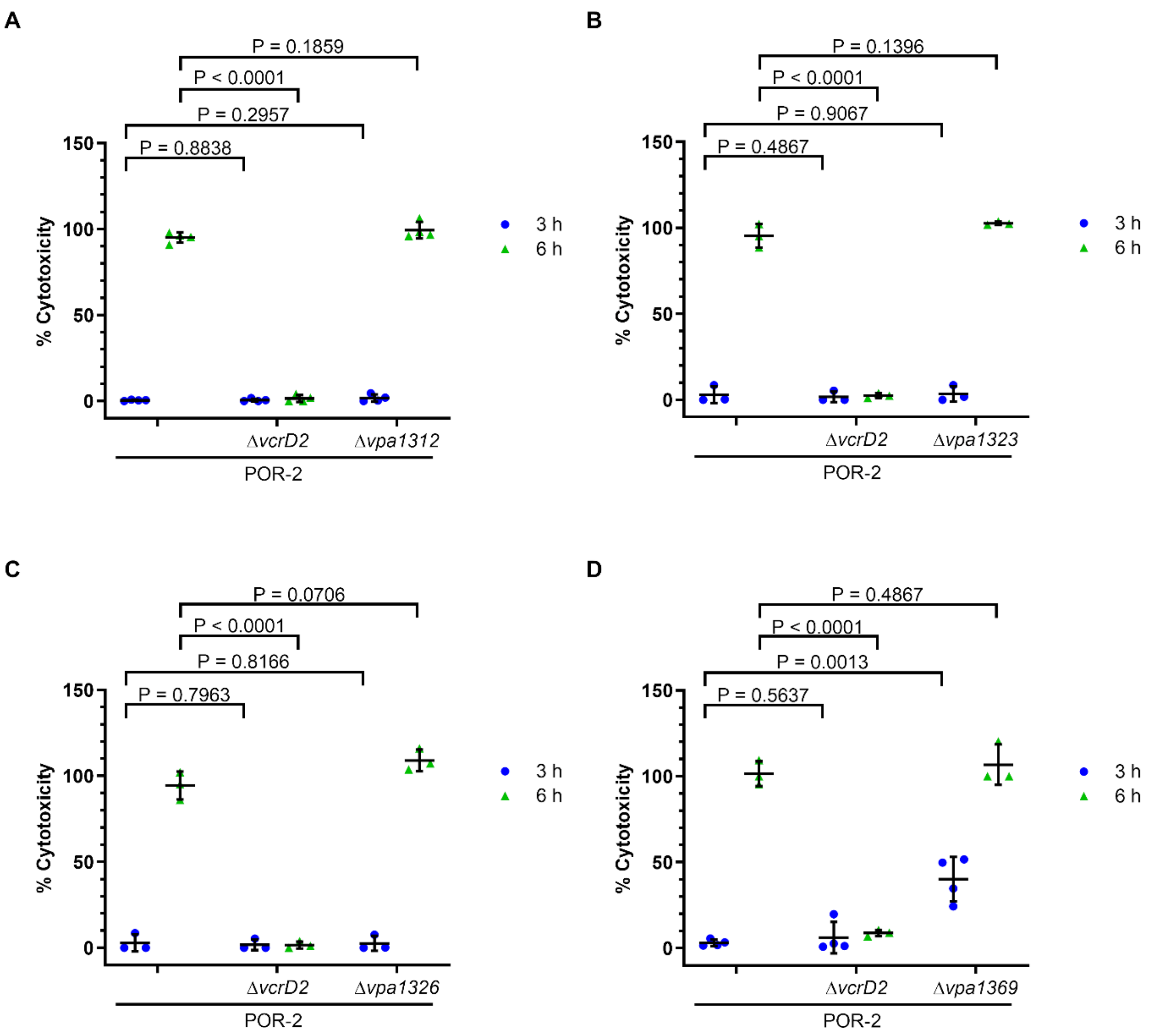

Fig. S2 Cytotoxic effect of *V. parahaemolyticus* lacking T3SS2-secreted proteins on Caco-2 cells. (A) *vpa1312* mutant, (B) *vpa1323* mutant, (C) *vpa1326* mutant and (D) *vpa1369* mutant. The bars represent the average of three independent experiments. The error bars indicate the SDs.

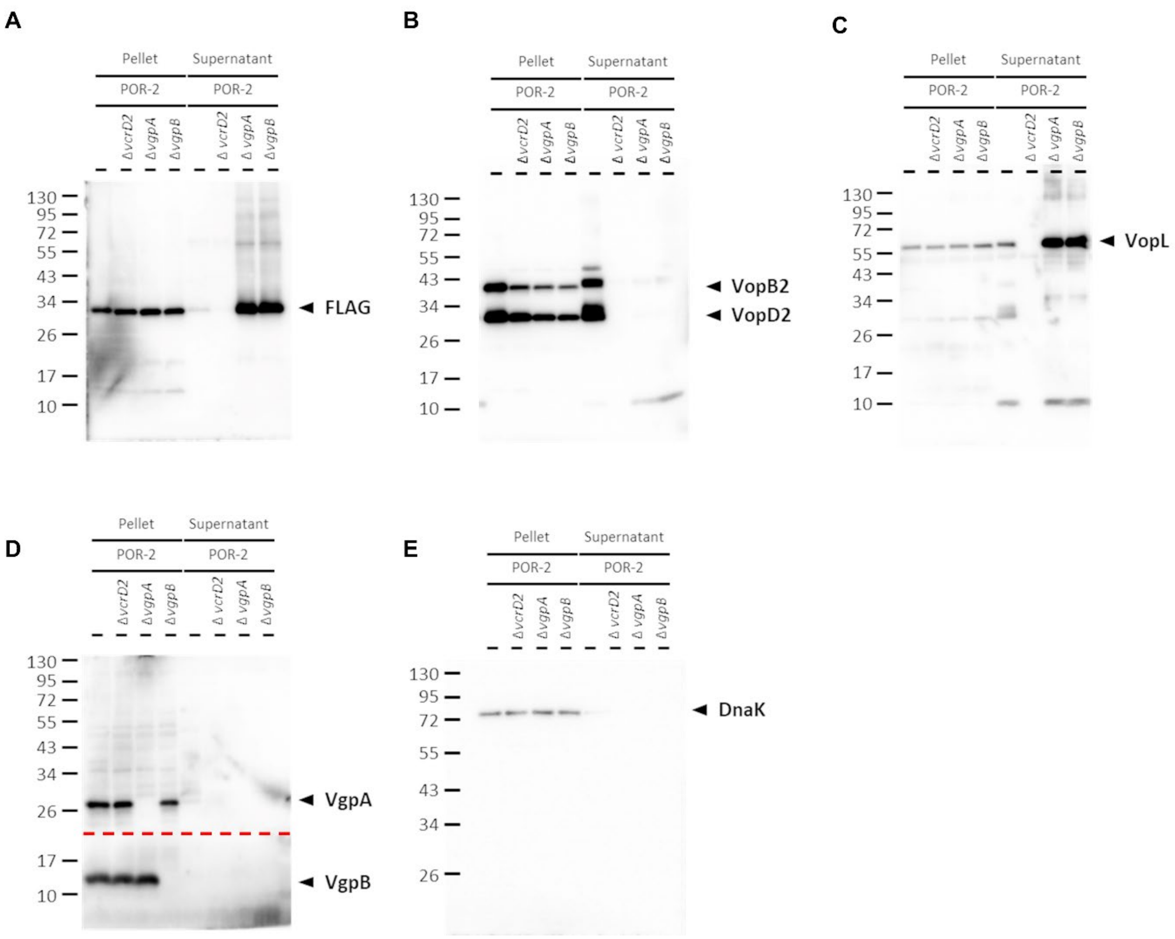

Fig. S3 Highly secretion of VPA1369 by gatekeeper mutants. Triple FLAG was tagged at the C-terminal of VPA1369 of *V. parahaemolyticus* POR-2 and derivative strain. Western blot analysis demonstrated the productions and secretions of VPA1369 with triple FLAG tag (A), VopB2 and VopD2 (B), VopL (C), VgpA and VgpB (D) by *V. parahaemolyticus* POR-2 and derivative strains cultured in LB broth with 0.04% crude bile. DnaK was used as a control for sample preparation (E). The figure was representative of three independent experiments. The red dashed line indicated the cutting point of the membrane before they separated to react with different primary antibodies.

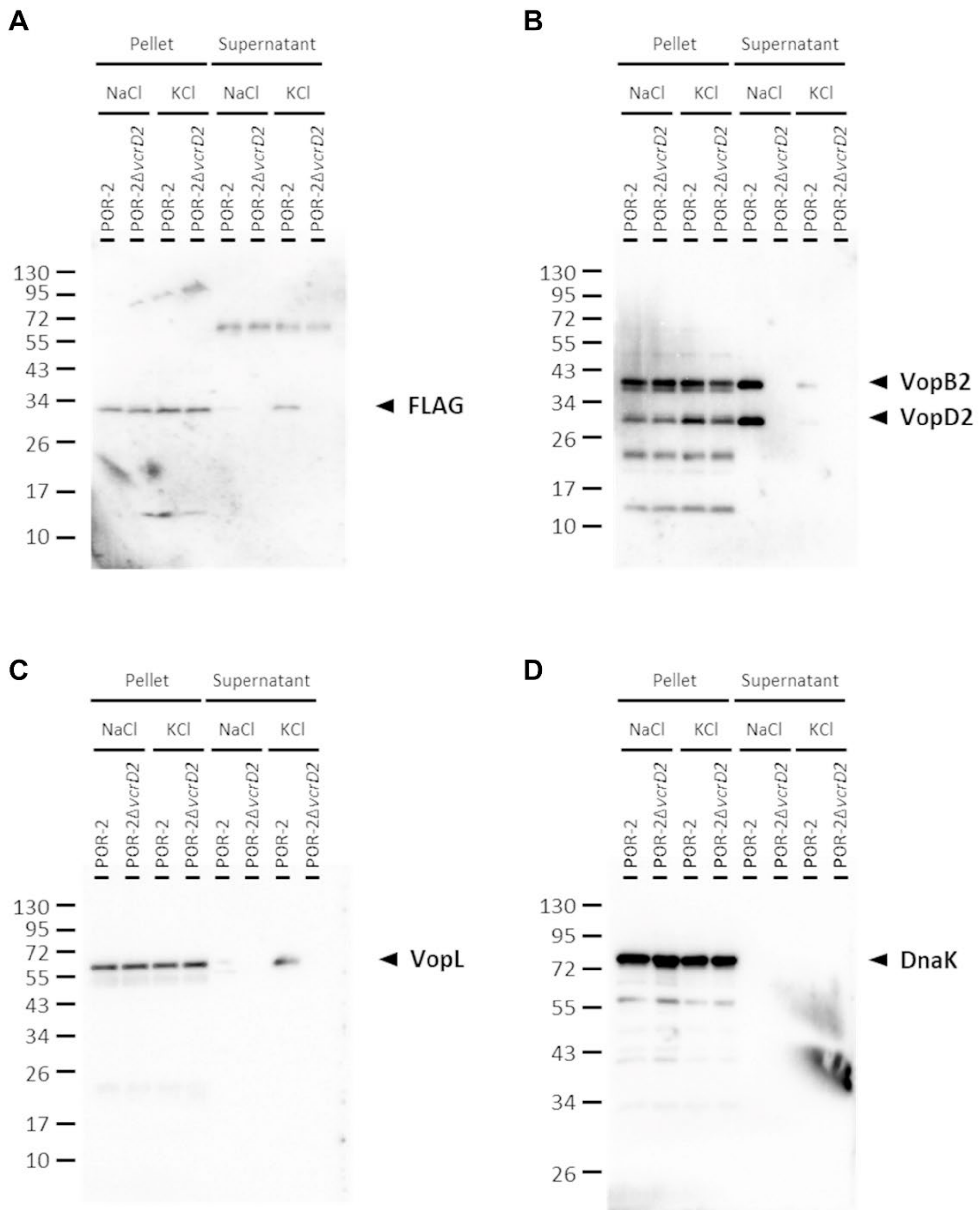

Fig. S4 Exposure of K^+ stimulates VPA1369 secretion. Triple FLAG was tagged at the C-terminal of VPA1369 of *V. parahaemolyticus* POR-2 and derivative strain. Western blot analysis demonstrated the productions and secretions of VPA1369 with triple FLAG tag (A), VopB2 and VopD2 (B), and VopL (C) by *V. parahaemolyticus* POR-2 and derivative strains cultured in LB broth with 0.1M NaCl or 0.1M KCl. DnaK was used as a control for sample preparation (D). The figure was representative of three independent experiments.

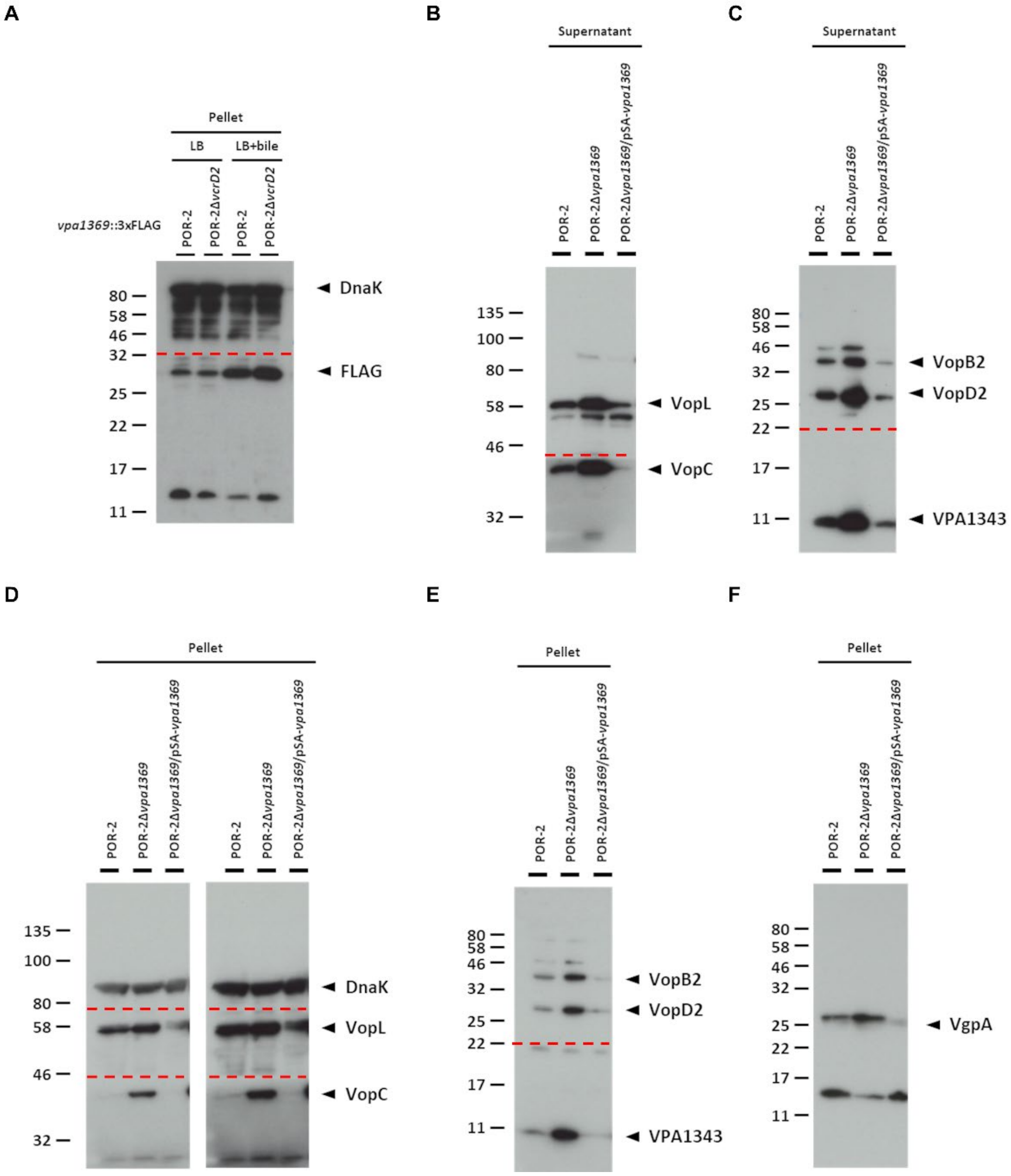

Fig. S5 Crude bile promotes VPA1369 production (A). Western blot analysis demonstrated the productions and secretions of T3SS2-related protein production and secretion (B-F). Western blot analysis demonstrated the secretions of VopL and VopC (B) and VopB2, VopD2 and VPA1343 (C). Western blot analysis demonstrated the production of VopL and VopC (D), VopB2, VopD2 and VPA1343 (E) and VgpA (F). DnaK was used as a control for sample preparation (D). The figure was representative of three independent experiments. The red dashed line indicated the cutting point of the membrane before they separated to react with different primary antibodies.

A

Gene		Fold changes (vs WT)		
		Δ*vgpA*	Δ*vgpB*	Δ*vtrN*
vp0819	*toxS*	1.1438	1.2179	-1.0322
vp0820	*toxR*	1.4233	1.1312	1.0301
vpa1332	*vtrA*	1.9406	1.7240	1.7538
vpa1333	*vtrC*	5.8866	5.9672	4.8386
vpa1348	*vtrB*	2.6320	2.6948	2.3932

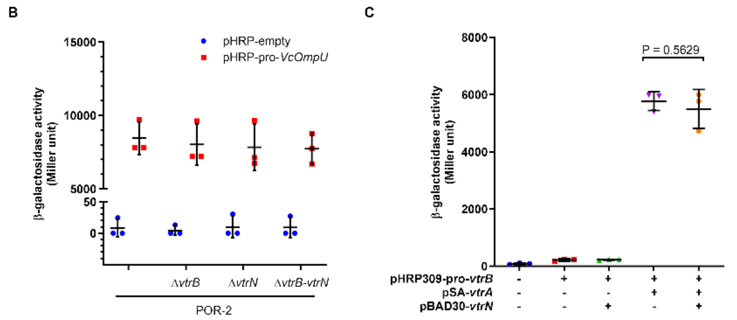

Fig. S6 VtrN inhibits VtrB-mediated T3SS2 transcriptional regulation. (A) Fold change in gene transcription of T3SS2 transcriptional regulation systems determined via RNA-seq. (B) β-Galactosidase activity of the β-galactosidase reporter gene under the *ompU* promoter (pHRP309-pro-*ompU*) in the *V. parahaemolyticus* strain POR-2 and derivative strains. The bars represent the average of three independent experiments. The error bars indicate the SDs. (C) β-Galactosidase activity from co-expressing VtrA (pSA-*vtrA*) and VtrN (pBAD30-*vtrN*) with a β-galactosidase reporter gene under the *vtrB* promoter (pHRP309-pro-*vtrB*) in *E. coli* strain MC4100 in LB broth with 0.02% arabinose. The bars represent the average of three independent experiments. The error bars indicate the SDs.
