# Supplementary material for: Regulatory mechanism for host-cell contact-dependent T3SS gene expression in Vibrio parahaemolyticus
Source: mSystems. 2025 Jun 17;10(7):e00251-25. doi: 10.1128/msystems.00251-25 (PMC12282083; doi:10.1128/msystems.00251-25)
Supplement: Supplemental captions — Captions for Tables S1 to S6. [file msystems.00251-25-s0002.pdf]

Supplementary table legends

Regulatory mechanism for host-cell contact-dependent T3SS gene expression in *Vibrio parahaemolyticus*

Sarunporn Tandhavanant[1,2], Hiroyuki Terashima[1,3], Hirotaka Hiyoshi[1], Dhira Saraswati Anggramukti[4], Nopadol Precha[2,5], Tetsuya Iida[4], Shigeaki Matsuda[4,] Narisara Chantratita[2], Toshio Kodama[1*]

[1]Department of Bacteriology, Institute of Tropical Medicine, Nagasaki University, Nagasaki, Japan

[2]Department of Microbiology and Immunology, Faculty of Tropical Medicine, Mahidol University, Bangkok, Thailand

[3]Department of Pharmacology, College of Pharmacy, Kinjo Gakuin University, Nagoya, Japan

[4]Department of Bacterial Infections, Research Institute for Microbial Diseases, Osaka University, Osaka, Japan

[5]Department of Environmental Health and Technology, School of Public Health, Walailak University, Nakhon Si Thammarat, Thailand

***Address correspondence to** Toshio Kodama, tkodama@nagasaki-u.ac.jp

Table S1 Gene transcription data from RNA-seq of *V. parahaemolyticus* RIMD2210633 (WT) and gatekeeper mutants (WTΔ*vgpA* and WTΔ*vgpB*)

Table S2 Spectrum counts of secreted proteins from proteomic analysis of *V. parahaemolyticus* POR-2Δ*vcrD2* and gatekeeper mutants

Table S3 Gene transcription data from RNA-seq of *V. parahaemolyticus* RIMD2210633 (WT) and WTΔ*vpa1369*

Table S4 List of bacteria and yeast in this study

Table S5 List of plasmids in this study

Table S6 List of primers and sequences in this study
